# Identification of LINC02310 as an enhancer in lung adenocarcinoma and investigation of its regulatory network via comprehensive analyses

**DOI:** 10.1186/s12920-020-00834-6

**Published:** 2020-12-11

**Authors:** Wenyuan Zhao, Jun Wang, Qingxi Luo, Wei Peng, Bin Li, Lei Wang, Chunfang Zhang, Chaojun Duan

**Affiliations:** 1 Department of Thoracic Surgery, Xiangya Hospital, Central South University, Changsha, Hunan People’s Republic of China; 2 Department of Oncology, Xiangya Hospital, Central South University, Changsha, Hunan People’s Republic of China; 3 Institute of Medical Sciences, Xiangya Hospital, Central South University, Changsha, Hunan People’s Republic of China; 4 National Clinical Research Center for Geriatric Disorders, Xiangya Hospital, Central South University, Changsha, Hunan People’s Republic of China

**Keywords:** LADC, LncRNA, TCGA, ceRNA regulatory network, PPI

## Abstract

**Background:**

Lung adenocarcinoma (LADC) is a major subtype of non-small cell lung cancer and has one of the highest mortality rates. An increasing number of long non-coding RNAs (LncRNAs) were reported to be associated with the occurrence and progression of LADC. Thus, it is necessary and reasonable to find new prognostic biomarkers for LADC among LncRNAs.

**Methods:**

Differential expression analysis, survival analysis, PCR experiments and clinical feature analysis were performed to screen out the LncRNA which was significantly related to LADC. Its role in LADC was verified by CCK-8 assay and colony. Furthermore, competing endogenous RNA (ceRNA) regulatory network construction, enrichment analysis and protein–protein interaction (PPI) network construction were performed to investigate the downstream regulatory network of the selected LncRNA.

**Results:**

A total of 2431 differentially expressed LncRNAs (DELncRNAs) and 2227 differentially expressed mRNAs (DEmRNAs) were from The Cancer Genome Atlas database. Survival analysis results indicated that lnc-YARS2-5, lnc-NPR3-2 and LINC02310 were significantly related to overall survival. Their overexpression indicated poor prognostic. PCR experiments and clinical feature analysis suggested that LINC02310 was significantly correlated with TNM-stage and T-stage. CCK-8 assay and colony formation assay demonstrated that LINC02310 acted as an enhancer in LADC. In addition, 3 targeted miRNAs of LINC02310 and 414 downstream DEmRNAs were predicted. The downstream DEmRNAs were then enriched in 405 Gene Ontology terms and 11 Kyoto Encyclopedia of Genes and Genomes pathways, which revealed their potential functions and mechanisms. The PPI network showed the interactions among the downstream DEmRNAs.

**Conclusions:**

This study verified LINC02310 as an enhancer in LADC and performed comprehensive analyses on its downstream regulatory network, which might benefit LADC prognoses and therapies.

## Background

NSCLC is a major cause of cancer-derived death worldwide, accounting for 85% of all lung cancers [1]. It can be classified into four subtypes: Lung adenocarcinoma (LADC), lung large cell carcinoma (LCLC), lung squamous cell carcinoma (LSCC) and lung neuroendocrine tumor (Lung NET) [2]. LADC, as a major type of NSCLC, has one of the highest mortality rates. Although LADC treatments, including surgery, chemotherapy, radiotherapy and other molecular target therapies have been improved, the long-term survival remains dim. Therefore, it is quite necessary to find new biomarkers for precise LADC treatments and predicting prognostic.

LncRNAs, which were previously considered as transcriptional noise, have been found to participate in regulatory biological processes such as transcriptional regulation, RNA processing and chromatin interaction. LncRNAs also participate in the stability and translation of mRNA and influence cell signaling [3, 4]. In addition, LncRNAs have been reported to promote LADC cell proliferation, invasion, metastasis and tumor progression, such as LncRNA NEAT1 [5], DGCR5 [6], H19 [7]. Moreover, previous studies showed that LncRNAs play an important role in cancer drug resistance [8–11]. Therefore, it is reasonable to find certain new prognostic biomarkers for LADC among LncRNAs.

In addition, LncRNAs were widely considered to function as ceRNAs, which regulate mRNA expression by sponging miRNAs in cancers [12–14]. These ceRNA regulatory networks constructed by coding and non-coding transcripts can result in several diseases when dysregulated [15]. Moreover, researches have demonstrated the significance of ceRNA regulatory network during the oncogenesis and progression of various cancers [16–19]. Some researchers have further identified potential prognostic mRNAs for LADC based on ceRNA regulatory network [20–29].

This study identified LINC02310 as an enhancer in LADC and investigated its regulatory network through bioinformatic analyses and experimental validation. Firstly, differential expression analyses based on the expression data from TCGA were performed to screen DELncRNAs and DEmRNAs. Next, the correlation p-values of overall survival and DELncRNA expression level in LADC patients were estimated by survival analysis. The top-3 significant DELncRNAs associated with overall survival were selected for further analysis. Then, PCR experiments and the relevant clinical feature analyses were conducted to evaluate the clinical correlation of the three DELncRNAs in LADC, among which LINC02310 was the most meaningful. Moreover, CCK-8 assays and colony formation assays were performed to demonstrate that LINC02310 acted as an enhancer in LADC. Furthermore, ceRNA regulatory network was constructed to find the targeted miRNAs and downstream DEmRNAs. In addition, GO functional annotation and KEGG pathway analyses of the downstream DEmRNAs provided a comprehension of their functions and mechanisms. Finally, PPI network was constructed to visualize the interactions among these downstream DEmRNAs.

## Methods

### LADC data sets and preprocessing

The LADC data sets, including RNA expression quantification data and the relevant clinical data, were downloaded from TCGA database. The expression quantification data of mRNAs and LncRNAs were obtained from HTSeq-counts files on Illumina platform. A total of 533 LADC samples and 59 adjacent normal samples were collected. The IDs of mRNAs and LncRNAs were converted using GENCODE Human Release 31.

### Differential expression analysis

DELncRNAs and DEmRNAs between LADC and adjacent normal samples were analyzed using the R package DESeq2 (version 1.22.2) [30]. Firstly, the samples were divided into two groups: LADC and adjacent normal samples. Next, the genes whose sum of expression is less than 20 were removed since too small gene expression can lead to large calculation errors. The DESeq function in the R package DESeq2 was then used to analyze the differential expression of the remaining genes and obtain the result dataset. Subsequently, the LncRNAs in the result data set were screened to obtain DELncRNAs according to the criteria of |log2 fold change (LFC)|> 1.5 and false discovery rate (FDR, or adjusted p-value) < 0.05. As for DEmRNAs, the criteria were |LFC|> 2 and FDR < 0.01. Finally, the R packages ggplot2 (version 3.1.1) and pheatmap (version 1.0.12) were used to plot the volcano plots and heatmaps of DELncRNAs and DEmRNAs.

### Survival analysis

To begin with, the samples with missing survival status information were removed from the expression data of DELncRNAs. Next, the R package DESeq2 was used to normalize the expression data of DELncRNAs. Next, the LADC samples were divided into two groups: high expression (normalized expression > median expression) and low expression (normalized expression < median expression) according to the expression level of the DELncRNAs in these samples. The R package survival (version 2.44-1.1) was then used to build the survival information dataset and to perform the log-rank test to calculate the correlation p-value of overall survival and DELncRNA expression level. The top-3 significant DELncRNAs related to overall survival were selected for further investigation. The survival curves of the three DELncRNAs were calculated with Kaplan–Meier (KM) method and visualized using the R package survminer (version 0.4.4). Lastly, the significance was calculated again by performing Fisher's accurate test and Pearson’s chi-squared test to validate the correlation between the overall survival and gene expression level.

### PCR experiments

LADC and adjacent normal tissues were obtained from 22 patients who suffered from LADC. These patients were treated in Xiangya Hospital, Central South University in 2015. All subjects had written consents, and the protocol was approved by the ethics committee of Xiangya Hospital (No. 201503303). All procedures conducted in the study were in accordance with the Ethics Standards Institutions Research Committee and the 1964 Helsinki Declaration and its subsequent amendments or similar ethical standards. All patients had no history of other malignancies and never received radiotherapy or chemotherapy. All tissue samples were immediately frozen in liquid nitrogen and stored at − 80 °C. All tumors and matched normal tissues were confirmed by two pathologists. Total RNA was extracted using the Trizol Reagent kit (Invitrogen) and was reversely transcribed into cDNAs by using HiScript II Q Select RT SuperMix for qPCR (Vazyme). Real-time PCR was performed on the cDNA templates using specific primers (TSINGKE) and the GeneCopoeia BlazeTaq™ SYBR® Green qPCR mix (GeneCopoeia). The LncRNAs relative expression levels were calculated as a ratio normalized to U6 expression. Comparative quantification was calculated with the 2^−ΔΔCt^ method. The primers sequences used in this study are listed. lnc-YARS2-5: F, 5′- ggtaccagaagcagcacct-3′; and R, 5′- aaaagaactcggccaagctc-3′. lnc-NPR3-2: F, 5′- aagcaagcatactcgtccct-3′; and R, 5′- gagccaagacgtagaggagg-3′. LINC02310: F, 5′-gaggaggtgctttgcttctc-3′; and R, 5′-atgaaccgagtcctggagtc-3'.

### Clinical feature analysis

The TNM-staging system has become the standard method for clinicians and medical scientists to stage malignant tumors. In this paper, we adopted its 8th edition, which is the latest revision proposed by International Association for the Study of Lung Cancer (IASLC) [31, 32]. T-stage represented the size and location of tumor, N-stage reflected the spread of lung cancer in lymph nodes, and M-stage indicated whether the cancer cell had metastasized to a distant site. They were explained detailly in Table 1. TNM-stage gave an overall evaluation of the tumor progression via integrating the information given by T-stage, N-stage and M-stage. Its stage I, II/III and IV represented the early, middle and advanced stages of tumor, respectively.

**Table 1:** Descriptions of T-stage, N-stage and M-stage

T-stage		N-stage		M-stage	
Stage	Description	Stage	Description	Stage	Description
T1	tumor size ≤ 3 cm	N0	No cancer cells near the lymph nodes	M0	Cancer cells have not metastasized
T2	3 cm < tumor size ≤ 5 cm	N1	Cancer cells have spread to the parabronchial nodes or the lymph nodes in the lungs of the primary tumor	M1	Cancer cells have metastasized
T3	5 cm < tumor size ≤ 7 cm	N2	Cancer cells have spread to the mediastinal lymph nodes	MX	Cancer metastasis is unknown
T4	tumor size > 7 cm	N3	Cancer cells have spread to lymph nodes without the primary tumor		
TX	undetectable	NX	Lymph nodes near the tumor were not tested		

The clinical feature analyses of the top-3 significant DELncRNAs were conducted based on the expression levels identified by PCR experiments and the clinical features of the 22 LADC patients. Firstly, the CT values of the experimental genes (DELncRNAs) and reference genes in LADC and adjacent normal samples were obtained from the PCR experimental results. Then, the ΔCT values were calculated as follows.

$$\begin{aligned} & \Delta {\text{CT}}_{{\text{LADC sample}}} = {\text{ CT}}_{{{\text{LADC sample}},{\text{ experimental gene}}}} - {\text{ CT}}_{{{\text{LADC sample}},{\text{ reference gene}}}} \\ & \Delta {\text{CT}}_{{\text{adjacent normal sample}}} = {\text{ CT}}_{{{\text{adjacent normal sample}},{\text{ experimental gene}}}} - {\text{ CT}}_{{{\text{adjacent normal sample}},{\text{ reference gene}}}} \\ \end{aligned}$$

The ΔΔCT values were calculated by

$$\Delta \Delta {\text{CT }} = \, \Delta {\text{CT}}_{{\text{LADC sample}}} - \, \Delta {\text{CT}}_{{\text{adjacent normal sample}}}$$

Let LFC = −ΔCT, and the LADC patients with |LFC|≤ 0.5 were removed. The remaining patients were separated into two groups: high expression (LFC > 0.5) and low expression (LFC < −0.5) according to the expression level of DELncRNAs in their tissues. Finally, the significance was calculated by performing Fisher's accurate test and Pearson’s chi-squared test to evaluate the correlation between the expression level of each of the three DELncRNAs and each clinical feature.

From the above analyses, LINC02310 can be screened out. Therefore, further validation of the clinical relevance of LINC02310 were performed based on the expression data and clinical data from TCGA. Firstly, LINC02310 expression data were extracted from TCGA. Then, the LADC samples were divided into two groups: high expression (LINC02310 expression > median expression) and low expression (LINC02310 expression < median expression). Lastly, the correlation significance between LINC02310 expression level and each clinical feature was evaluated by performing Fisher's accurate test and Pearson’s chi-squared test.

### CCK-8 assay and colony formation assay

Two human lung cancer cell lines (H1299, PC-9) were used in the CCK-8 assay and colony formation assay since they can be efficiently infected according to our previous studies. They were purchased from Chinese Academy of Sciences Cell Bank (Shanghai, China).

OE: the primers G0157014-1F (AGGATCGCTAGCGCTACCGGACTCAGATCTCGAGAGTCTCCTCTTG CAGATCAGATACCACC) and G0157014-1R (TACCCGGTAGAATTATCTAGAGTCGCGGGATCCAGATAC CTAAAAGGCACAAATCCTTCTG) were synthesized. They were then cloned into pLVX-Puro at XhoI-BamHI site to obtain the LINC02310 overexpression vector, pLVX-Puro-LINC02310, which was transfected into the cells.

NC: a random sequence was cloned into pLVX-Puro at XhoI-BamHI site to obtain the control vector, which was then transfected into the cells.

Si-02310: three small interfering RNAs (siRNAs) of LINC02310 were synthesized and transfected into the cells to down-regulate LINC02310 expression.

Si-NC: A control sequence was transfected into the cells.

CCK-8 assay: 100 μL of OE, NC, si-02310 and si-NC of H1299 and PC-9 (1 × 10^3^/mL) were transplanted in 96-well plate. Next, 10 μL of CCK-8 solution (Dojindo Laboratories, Kumamoto, Japan) was added to each well. Then, 40 min of incubation at 37 °C was involved. Finally, the absorbance was measured at 450 nm (OD450) by automatic microplate reader (Tecan, NANOQUANT, Swizerland) on days 0, 1, 2, 3 and 4, respectively. The experiment was repeated three times.

Colony formation assay: OE, NC, si-02310 and si-NC of H1299 (1 × 10^3^) were transplanted into 6-well plate and then incubated at 37 °C for 10 days. Colonies were dyed with dyeing solution containing 0.1% crystal violet and 20% methanol. Cell colonies were then counted and analyzed.

### CeRNA regulatory network construction

The lncRNASNP2 website [33, 34] was used to predict the targeted miRNAs of LINC02310. The targeted miRNAs whose reads of exon model per million mapped reads (RPM) ≥ 1 were screened out. The starBase database (version 3.0) [35] was used to predict the downstream mRNAs of the selected targeted miRNAs. The prediction programs TargetScan, microT, miRanda, miRmap, PITA, RNA22 and PicTar were integrated into starBase. Then, these downstream mRNAs were intersected with the DEmRNAs to get the downstream DEmRNAs. Finally, the ceRNA (LINC02310-targeted miRNAs-downstream DEmRNAs) regulatory network was constructed and visualized by the software Cytoscape (version 3.7.1).

### Enrichment analysis

The enrichment analysis consisted of Gene Ontology (GO) functional annotation and Kyoto Encyclopedia of Genes and Genomes (KEGG) pathway analysis. It was performed using the R packages clusterProfiler (version 3.10.1) [36] and org.Hs.eg.db (version 3.7.0), which provided analysis functions and human gene annotation information, respectively. They were used to analyze and visualize the functional profiles of the predicted downstream DEmRNAs.

The GO functional annotation was performed, including molecular function (MF), biological process (BP) and cellular component (CC) [37]. Firstly, the gene IDs of downstream DEmRNAs were listed. The enrichGO function was then used to generate the enrichment result data set of MF, BP and CC, respectively. Finally, the barplot function was used to visualize the result.

In addition, the KEGG pathway analysis was conducted. Firstly, the gene IDs of downstream DEmRNAs were converted into a format suitable for KEGG pathway analysis according to HGNC database. Secondly, the enrichKEGG function was used to generate KEGG pathway analysis result data set. Lastly, the result was visualized via barplot function.

### PPI network

The PPI network was constructed using the STRING database (version 11.0) and Cytoscape according to the following steps. First, the gene names of downstream DEmRNAs were uploaded to the STRING database to build a PPI dataset. In the dataset, the relevance between every two proteins (nodes) were evaluated by a combined score. The score ranges from 0 to 1, and a higher score represents a closer interaction. Second, the interactions whose combined score > 0.7 were screened out and downloaded. Finally, the downloaded interactions were divided into different modules using MCODE (version 1.5, a plugin for Cytoscape). The module with the highest average combined score was selected to construct the PPI network, which was visualized by Cytoscape.

## Results

### DELncRNAs and DEmRNAs in LADC

**Fig. 1:**
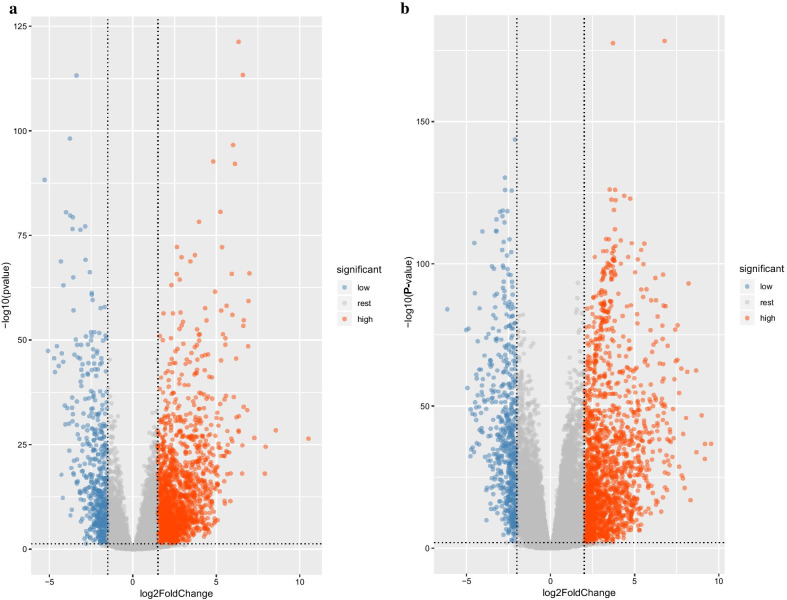
Volcano plots of LncRNAs and mRNAs. **a** LncRNAs: the red dots represent the LncRNAs with LFC > 1.5 and *p* < 0.05; the blue dots represent the LncRNAs with LFC < -1.5 and *p* < 0.05; the gray dots are the remaining LncRNAs; **b** mRNAs: the red dots represent the mRNAs with LFC > 2 and *p* < 0.01; the blue dots represent the mRNAs with LFC < -2 and *p* < 0.01; the gray dots are the remaining mRNAs

**Fig. 2:**
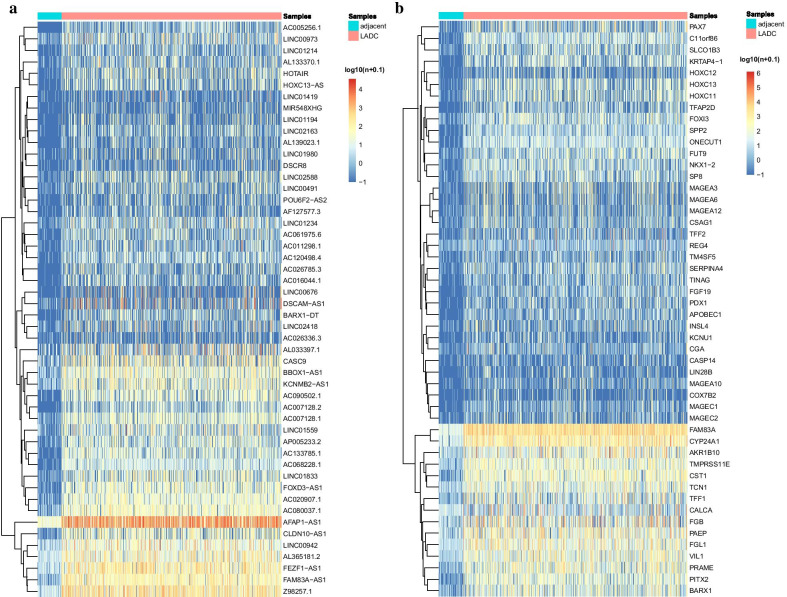
Heatmaps of the top-50 DELncRNAs and DEmRNAs. **a** DELncRNAs; **b** DEmRNAs. The samples are divided into two groups: LADC and adjacent normal samples. The ‘*n*’ in ‘log10(*n* + 0.1)’ is the normalized read count (expression quantity), and adding 0.1 is to avoid meaningless calculation when *n* = 0

The expression data of 12,459 LncRNAs and 19,647 mRNAs in LADC and adjacent normal samples were extracted from TCGA database. The differential expression analysis results of these LncRNAs and mRNAs were visualized in the volcano plots (Fig. 1), respectively. The red dots and blue dots represent the significantly high-expressed RNAs and low-expressed RNAs, respectively, and the gray dots are the remaining RNAs. As a result, 2431 DELncRNAs (Additional file 1: Table S1, 1931 high-expressed and 500 low-expressed) and 2227 DEmRNAs (Additional file 2: Table S2, high-expressed and 570 low-expressed) were identified. The expression of the top-50 DELncRNAs and DEmRNAs in LADC and adjacent normal samples were visualized in the heatmaps (Fig. 2), respectively. Significant difference can be seen in the expression of each RNA in the two groups.

### Survival analysis of the DELncRNAs

**Fig. 3:**
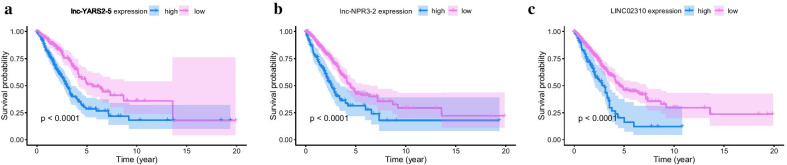
KM survival plots of the top-3 significant DELncRNAs. **a** lnc-YARS2-5 (Symbol ID: AC087588.2, *p* = 6.9774e−7); **b** lnc-NPR3-2 (Symbol ID: AC034223.1, *p* = 5.2233e−6); **c** LINC02310 (*p* = 1.0380e−5)

Setting *p* < 0.05 as a cutoff, 389 DELncRNAs were identified as significantly related to overall survival (Additional file 3: Table S3). Lnc-YARS2-5, lnc-NPR3-2 and LINC02310 were the top-3 significant DELncRNAs related to overall survival. The KM survival plots of the three DELncRNAs were shown in Fig. 3. It can be observed that high expression of the three DELncRNAs lead to poor survival probabilities. In Table 2, p1 and p2 were the correlation p-values of Fisher’s accurate test and Pearson’s chi-squared test, respectively. The results further confirmed that these three DElncRNAs were significantly related to overall survival.

**Table 2:** Validation of the correlation significance between the overall survival and gene expression level

Status	lnc-YARS2-5				lnc-NPR3-2				LINC02310			
	Low	High	*p*_1_	*p*_2_	Low	High	*p*_1_	*p*_2_	Low	High	*p*_1_	*p*_2_
Dead	66	117	3.28e−6	2.31e−6	117	66	7.77e−5	4.76e−5	126	57	3.54e−4	2.74e−4
Alive	186	135			258	63			266	55		

‘Low’ and ‘high’ represent the LADC samples from TCGA in which the DELncRNAs were low expressed and high expressed, respectively. *p*_1_ and *p*_2_ are the correlation p-values of Fisher’s accurate test and Pearson’s chi-squared test, respectively

### PCR experiments and clinical feature analyses

**Fig. 4:**
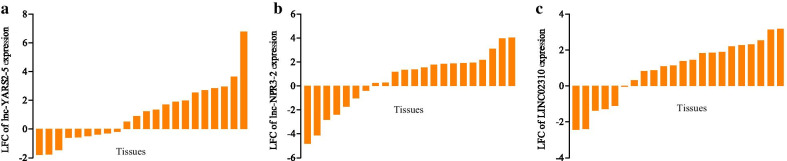
PCR expression quantification in LADC tissues. **a** lnc-YARS2-5; **b** lnc-NPR3-2; **c** LINC02310

Lnc-YARS2-5, lnc-NPR3-2 and LINC02310 were further investigated by PCR experiments based on the expression level in LADC tissues and its related clinical features. The expression quantification (Fig. 4) were obtained with the 2^−ΔΔCt^ method and showed that the three DELncRNAs were mainly up-regulated in the LADC tissues, which was consistent with the survival analysis results.

Table 3 showed the clinical feature analyses of the three DELncRNAs by performing Fisher's accurate test (*p*_1_) and Pearson’s chi-squared test (*p*_2_), respectively. These DELncRNAs were correlated with clinical T-stage and TNM-stage in LADC tissues from Xiangya Hospital. It can be observed that LINC02310 had the most significant effect on tumor proliferation. This result was further validated through the clinical analysis of LINC02310 based on TCGA database, as shown in Table 4. Both Fisher's accurate test (*p*_1_) and Pearson’s chi-squared test (*p*_2_) demonstrated that LINC02310 expression was significantly related to TNM-stage, T-stage and N-stage. Based on the above results, LINC02310 was screened out and reasonably assumed to act as an enhancer in LADC.

**Table 3:** LncRNA expression level and clinical feature analysis of 22 cases of LADC from Xiangya hospital

Clinical features		lnc-YARS2-5				lnc-NPR3-2				LINC02310			
		Low	High	*p*_1_	*p*_2_	Low	High	*p*_1_	*p*_2_	Low	High	*p*_1_	*p*_2_
Age													
< 55		1	5	0.615	0.457	3	5	> 0.99	0.636	2	6	> 0.99	> 0.99
≥ 55		4	8			3	8			3	9		
Gender													
Male		4	8	0.615	0.457	6	7	0.109	*0.044*	5	9	0.260	0.091
Female		1	5			0	6			0	6		
T-stage													
T1		5	4	*0.029*	*0.009*	1	8	0.141	0.069	0	10	*0.033*	*0.010*
T2/T3/T4		0	9			5	5			5	5		
N-stage													
N0		5	7	0.114	0.063	1	10	*0.041*	*0.013*	2	11	0.290	0.176
N1/N2/N3		0	6			5	3			3	4		
TNM-stage													
I		4	4	0.118	0.060	1	7	0.177	0.127	0	9	*0.038*	*0.020*
II/III/IV		1	9			5	6			5	6		
Differentiation													
Low		1	5	0.542	0.395	2	4	> 0.99	0.937	2	4	0.346	0.310
Mid-low		1	2			1	2			1	2		
Mid		1	3			2	2			0	4		
Mid-high		1	0			1	1			2	1		

‘Low’ and ‘high’ denote the cases where the DElncRNAs were low expressed and high expressed, respectively. *p*_1_ and *p*_2_ are the p-values obtained via Fisher’s accurate test and Pearson’s chi-squared test, respectively

**Table 4:** Clinical feature analysis of LINC02310 based on TCGA clinical data

Clinical features	Low	High	*p*_1_	*p*_2_
Gender				
Female	216	60	0.831	0.776
Male	183	54		
Race				
American Indian or Alaska native	1	0	0.762	0.745
Asian	6	1		
Black or African American	43	9		
White	300	87		
Age				
< 55	56	15	0.878	0.803
≥ 55	328	95		
TNM-stage				
I	222	52	*0.033*	*0.027*
II	95	26		
III	55	29		
IV	21	5		
T-stage				
T1/T2	352	92	*0.043*	*0.038*
T3/T4/TX	47	22		
N-stage				
N0	268	62	*0.035*	*0.035*
N1	71	24		
N2/N3/NX	60	27		
M-stage				
M0	265	79	0.726	0.655
M1	21	4		
MX	111	29		
Pack-years smoked				
< 20	49	10	0.389	0.310
≥ 20	225	67		

‘Low’ and ‘high’ represent the number of LADC samples where LINC02310 were low expressed and high expressed, respectively. *p*_1_ and *p*_2_ are the p-values of Fisher’s accurate test and Pearson’s chi-squared test, respectively

### CCK-8 assay and colony formation assay

**Fig. 5:**
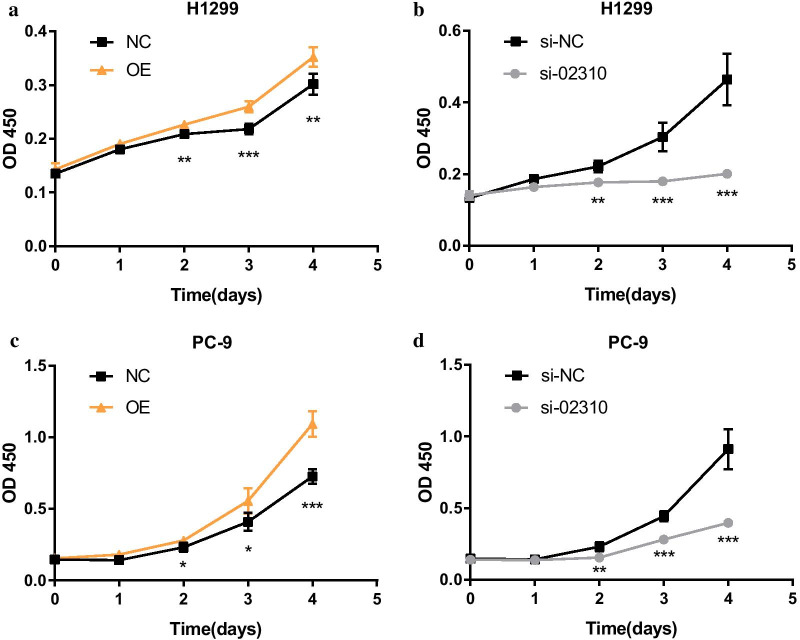
CCK-8 assays. LINC02310 promoted the cell proliferation of H1299 and PC-9. **a** H1299 OE/NC; **b** H1299 si-02310/si-NC; **c** PC-9 OE/NC; **d** PC-9 si-02310/si-NC. *p < 0.05, **p < 0.01, ***p < 0.001

**Fig. 6:**
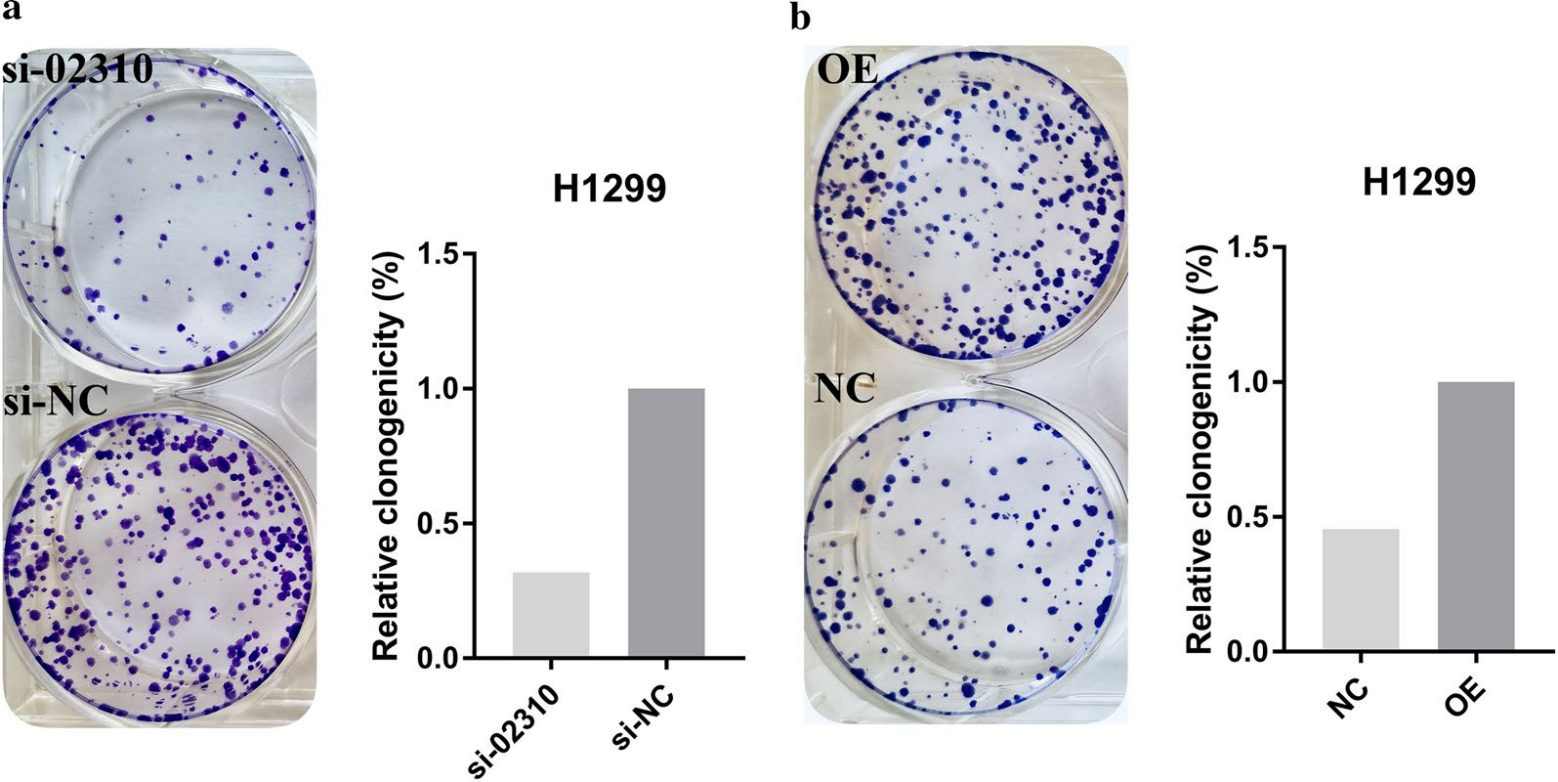
Colony formation assays. LINC02310 promoted the clone formation of H1299 cells. **a** H1299 si-02310/si-NC; **b** H1299 OE/NC

CCK-8 assay and colony formation assay were performed to verify the assumption that LINC02310 promotes cell proliferation in LADC. Firstly, the linear transcript expression vector was constructed. Next, OE, NC, SI and SI-NC were transfected into H1299 and PC-9 cell lines, respectively. Subsequently, the effect of LINC02310 on cell proliferation was examined. CCK-8 assays showed that forced expression of LINC02310 enhanced proliferation of H1299 and PC-9 (Fig. 5). Similarly, colony formation assay indicated that H1299 cell proliferation was significantly increased by forced expression of LINC02310 (Fig. 6). It can be concluded that LINC02310 overexpression significantly promoted cell proliferation and depressing LINC02310 expression conspicuously inhibited cell proliferation.

### CeRNA regulatory network construction

**Fig. 7:**
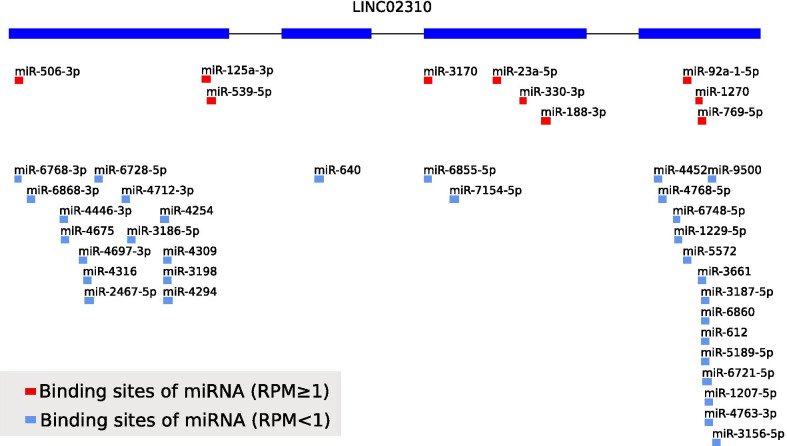
Binding sites of miRNAs with LINC02310. The red blocks and light blue blocks represent targeted miRNAs with RPM ≥ 1 and RPM < 1, respectively

**Fig. 8:**
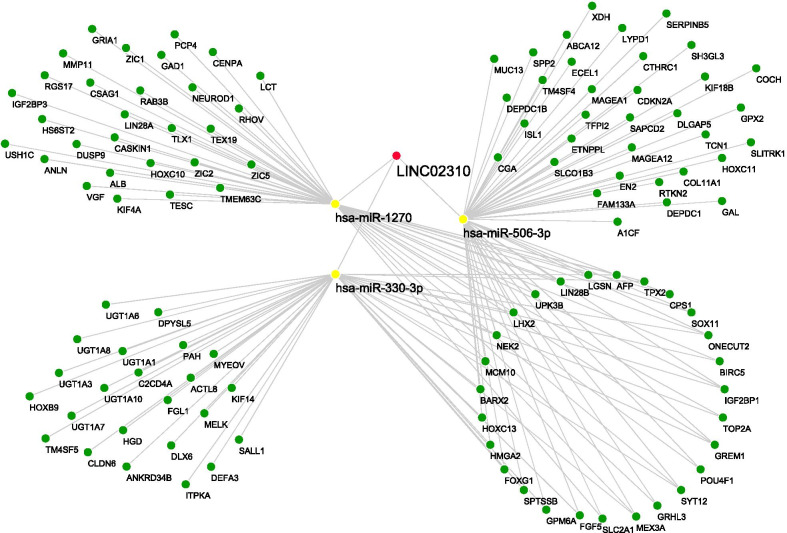
CeRNA (LINC02310-targeted miRNAs-downstream DEmRNAs) regulatory network. Red dot: LINC02310; yellow dots: targeted miRNAs; green dots: downstream DEmRNAs. downstream DEmRNAs connected to more than one targeted miRNA were placed together at the lower right in a circle

MiRNA target sites on LINC02310 were illustrated in Fig. 7 according to LncRNASNP2 database. The targeted miRNAs with RPM ≥ 1 were selected to interact with 2227 DEmRNAs via starBase. As a result, has-miR-1270, has-miR-506-3p and has-miR-330-3p were involved in 561 interactions with 414 downstream DEmRNAs (Additional file 4: Table S4). The downstream DEmRNAs with |LFC|> 3.5 and FDR < 0.01 were further selected from these interactions to construct the ceRNA regulatory network (Fig. 8) using Cytoscape. The ceRNA regulatory network contained 117 nodes and 149 edges with LINC02310, 3 targeted miRNAs and 113 downstream DEmRNAs involved.

### GO functional annotation and KEGG pathway analysis

**Fig. 9:**
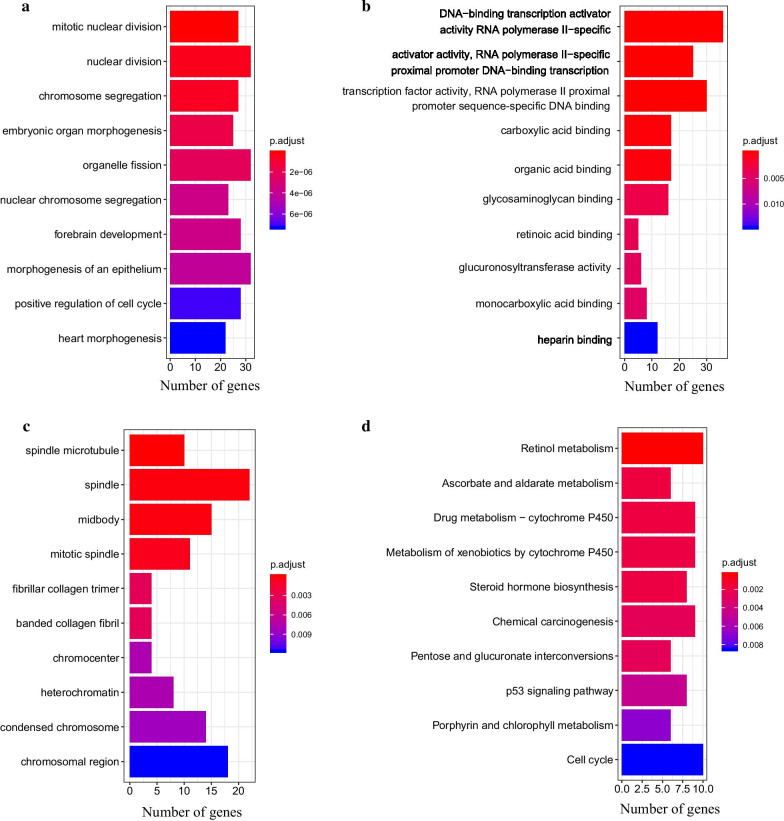
The top-10 significant in GO and KEGG pathway analysis of the downstream DEmRNAs. **a** BPs; **b** MFs; **c** CCs; **d** KEGG pathway

GO analysis result (Additional files 5–7: Tables S5–S7) showed that the 414 downstream DEmRNAs were significantly related to 405 GO terms (361 BPs, 17 MFs and 27 CCs) with p-value < 0.05. The top-10 significantly enriched GO terms were shown in Fig. 9. Most of the downstream DEmRNAs enriched in BPs (Fig. 9a) took part in nuclear division, chromosome segregation, morphogenesis, organelle fission, forebrain development and positive regulation of cell cycle. MF result (Fig. 9b) indicated that the downstream DEmRNAs were significantly associated with DNA binding activities. In addition, the most enriched CC was spindle (Fig. 9c). KEGG pathway analysis (Fig. 9d and Additional file 8: Table S8) showed that 11 pathways were significantly enriched with p-value < 0.05, which indicated that the downstream DEmRNAs were conspicuously associated with metabolism activities.

### Protein–protein interaction network analysis

**Fig. 10:**
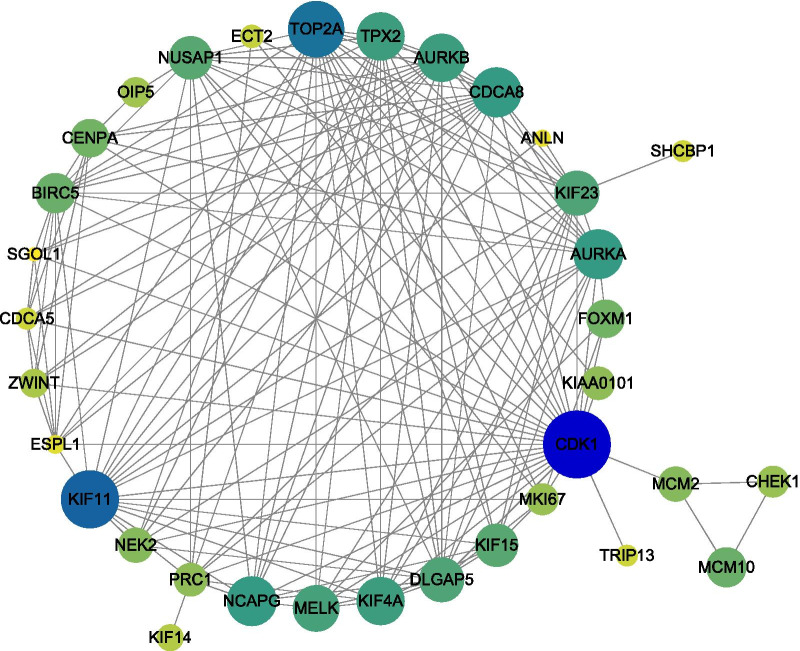
PPI network. The size and color of the nodes represent the degree, which is the number of nodes with which one node is interacted among the total 1081 interactions. Each line represents an interaction

A total of 1081 interactions (Additional file 9: Table S9) involving 226 nodes (downstream DEmRNAs) were identified using the STRING database with combined score > 0.7. These interactions were analyzed and divided into different modules using MCODE. The module with the highest combined score were screened out (Additional file 10: Table S10, 35 nodes and 534 interactions). For better visualization, these interactions were further screened with combined score > 0.95 to construct the PPI network (Fig. 10, 34 nodes and 156 interactions). The three downstream DEmRNAs with the highest degree are CDK1 (degree = 57), KIF11 (degree = 51) and TOP2A (degree = 50).

## Discussion

LADC is a major subtype of NSCLC and has a very high mortality rate [38]. The best opportunity for surgery is often lost because the early symptoms of LADC are insipid and the prognosis is still unsatisfactory. Thus, it is quite necessary to provide prognostic biomarkers for LADC treatments. With the development of molecular biology, LncRNA has been found to be related to the regulation of gene expression. Its ectopic expression can interact with DNA, proteins and RNA, leading to malignant transformation of cells, which can lead to a variety of cancers.

This study identified LINC02310 as an enhancer and promising biomarker in LADC via comprehensive analyses of TCGA data and experimental validation. LINC02310 has almost not been reported except by Yan‐Yan Li et al. [39] up until now. However, they simply identified LINC02310 as one of the ten prognostic markers in LADC through a Cox regression model. This study provided comprehensive bioinformatic analyses and further experiments to identify the significance of LINC02310. Further investigations about its targeted miRNAs and downstream DEmRNAs were performed.

To begin with, a total of 2431 DELncRNAs and 2227 DEmRNAs were identified. Next, lnc-YARS2-5, lnc-NPR3-2 and LINC02310 were identified as the top-3 significant DELncRNAs associated with overall survival, and their high expression means low survival probability. The PCR experimental results also showed that these three DELncRNAs were up-regulated in most of the 22 pairs of LADC tissues from Xiangya Hospital, which was in accordance with the survival analysis. The clinical feature analysis of the 22 pairs of LADC tissues showed that LINC02310 was significantly associated with T-stage and TNM-stage in LADC, which was further verified by analysis of TCGA clinical data. These results indicated that LINC02310 acted as an enhancer in LADC. The results of CCK-8 assay and colony formation assay suggested that up-regulating LINC02310 significantly promoted the growth and proliferation of the LADC cells PC-9 and H1299. On the contrary, after down-regulating the expression of LINC02310, the growth and proliferation of the two cell lines were inhibited. In conclusion, LINC02310 was identified as an enhancer in LADC in this study.

In addition, it is necessary to investigate the downstream RNAs of LINC02310 to reveal the potential functions and mechanisms involved in the regulating pathways. 3 targeted miRNAs (has-miR-1270, has-miR-506-3p and has-miR-330-3p) and 414 downstream DEmRNAs were predicted via the lncRNASNP2 website and starBase database. Has-miR-1270 was reported to interacted with circRNAs to regulate the tumorigenesis and development of LADC [40] and the cell invasion and migration in NSCLC [41]. Has-miR-506-3p was identified as a tumor suppressor in several cancers, including NSCLC [42] and colorectal cancer [43]. It can also reverse gefitinib resistance in NSCLC by targeting Yes-associated protein 1 [44]. Has-miR-330-3p promoted cell invasion and metastasis [45] and controlled cell proliferation [46] in NSCLC. In conclusion, these three targeted miRNAs played an important role in NSCLC. However, they were rarely reported to interact with LncRNAs in lung cancer. 113 downstream DEmRNAs with |LFC|> 3.5 and FDR < 0.01 were further screened out to construct the ceRNA regulatory network, which revealed the interactions of LINC02310 with its 3 targeted miRNAs and downstream DEmRNAs.

GO and KEGG pathway analysis provided a further understanding of the functions and mechanisms associated with the downstream DEmRNAs. The top-10 GO terms and KEGG pathways were visualized, most of which were reported to associate with cancers. For example, nuclear division, which was the most significant BP term, is associated with promoting malignant progression in colorectal cancer [47]. Transcription factors play important roles in various cancers [48–50]. More potential biomarkers for LADC may be found by investigating these downstream DEmRNAs.

Furthermore, the PPI network of the downstream DEmRNAs was constructed and the three downstream DEmRNAs of the highest degrees were obtained, including CDK1, TOP2A and KIF11. CDK1 was reported to regulate the development of lung cancer by interacting with LncRNA [51], miRNA [52] and circRNA [53]. It also served as a prognostic biomarker for lung cancer [54]. The prognostic significance of TOP2A in NSCLC was revealed by bioinformatic analysis [55]. In addition, the mutations of TOP2A are associated with chemotherapy resistance in lung cancer [56]. KIF11 has been identified as potential biomarkers for NSCLC by several studies [57, 58]. These findings validated the significance of the downstream regulatory network analysis of LINC02310 in this study.

In summary, this study verified that LINC02310 significantly promoted the growth and proliferation of LADC cells, and was closely related to the clinical features of LADC patients. The regulatory network of LINC02310 in the carcinogenesis of LADC was further investigated, which may benefit future studies. However, to reveal the specific mechanisms of LINC02310 still requires a lot of experiments. The tumorigenesis and progression of LADC is a complex process regulated by multiple factors. In order to realize the early diagnosis of LADC and improve the prognosis of patients, we still need to investigate its potential regulatory mechanisms, which can improve the current diagnostic and treatment methods.

## Conclusions

In this study, LINC02310, which has rarely been investigated previously, was identified as an enhancer in LADC through bioinformatic analyses and experiments, including PCR experiment, CCK-8 assays and colony formation assays. In addition, the ceRNA regulatory network was constructed to identify the targeted miRNAs of LINC02310 and downstream DEmRNAs. GO functional annotation and KEGG pathway analyses showed the functions and mechanisms of the downstream DEmRNAs. The PPI network revealed the interactions among the downstream DEmRNAs. In conclusion, this research identified LINC02310 as a promising prognostic biomarker for LADC therapies and provided comprehensive analyses on its regulatory network.

## Supplementary information

**Additional file 1**.** Table S1**: List of DELncRNAs.

**Additional file 2**.** Table S2**: List of DEmRNAs.

**Additional file 3**.** Table S3**: 389 DELncRNAs significantly related to overall survival.

**Additional file 4**.** Table S4**: targeted miRNAs interacted with DEmRNAs.

**Additional file 5**.** Table S5**: GO analysis result (BP terms).

**Additional file 6**.** Table S6**: GO analysis result (MF terms).

**Additional file 7**.** Table S7**. GO analysis result (CC terms).

**Additional file 8**.** Table S8**: KEGG analysis result.

**Additional file 9**.** Table S9**: downstream DEmRNAs interactions.

**Additional file 10**.** Table S10**: PPI network clustered by MCODE.

## Data Availability

The LADC data sets, the expression quantification data of RNAs, and the relevant clinical datasets are available in TCGA database, (https://portal.gdc.cancer.gov/). GENCODE Human Release 31 (GRCh38.p12) was obtained from GENCODE, (https://www.gencodegenes.org/human/). The R packages used in this study were downloaded from Bioconductor, (http://www.bioconductor.org/packages/release/bioc/). The clinical data of 22 LADC patients and the raw data of PCR experiments are available in figshare website, (https://doi.org/10.6084/m9.figshare.13266500). Other data and materials are available as follows: LncRNASNP2 website, (http://bioinfo.life.hust.edu.cn/lncRNASNP#!/); StarBase database, (http://starbase.sysu.edu.cn/); the software Cytoscape, (https://cytoscape.org/download.html); HGNC database, (https://www.genenames.org/); STRING database, (https://string-db.org/); MCODE, (http://apps.cytoscape.org/apps/mcode).

## Abbreviations

LADC: Lung adenocarcinoma
NSCLC: Non-small cell lung cancer
LSCC: Lung squamous cell carcinoma
LCLC: Lung large cell carcinoma
TCGA: The Cancer Genome Atlas
DEmRNA: Differentially expressed mRNA
DELncRNA: Differentially expressed LncRNA
KM: Kaplan–Meier
ceRNA: Competing endogenous RNA
GO: Gene ontology
MF: Molecular function
BP: Biological process
CC: Cellular component
KEGG: Kyoto Encyclopedia of Genes and Genomes
PPI: Protein–protein interaction
